# Supplementary material for: Acrolein Promotes Aging and Oxidative Stress via the Stress Response Factor DAF-16/FOXO in Caenorhabditis elegans
Source: Foods. 2022 May 28;11(11):1590. doi: 10.3390/foods11111590 (PMC9180825; doi:10.3390/foods11111590)
Supplement: Supplementary file 1 [file foods-11-01590-s001.zip › foods-1698686-supplementary.pdf]

Table S1. Primer sequences for qRT-PCR.

Gene	Primer	Sequence (5′-3′)
daf-16	Forward	TTTCCGTCCCCGAACTCAA
	Reverse	ATTCGCCAACCCATGATGG
ctl-1	Forward	GCGGATACCGTACTCGTGAT
	Reverse	GTGGCTGCTCGTAGTTGTGA
ctl-2	Forward	TCCGTGACCCTATCCACTTC
	Reverse	TGGGATCCGTATCCATTCAT
